# Carrot *AOX2a* Transcript Profile Responds to Growth and Chilling Exposure

**DOI:** 10.3390/plants10112369

**Published:** 2021-11-03

**Authors:** Maria Doroteia Campos, Catarina Campos, Amaia Nogales, Hélia Cardoso

**Affiliations:** 1 MED—Mediterranean Institute for Agriculture, Environment and Development, Instituto de Investigação e Formação Avançada, Universidade de Évora, Pólo da Mitra, Ap. 94, 7006-554 Évora, Portugal; mccampos@uevora.pt (C.C.); anogales@isa.ulisboa.pt (A.N.); 2 LEAF—Linking Landscape, Environment, Agriculture and Food Research Center, Instituto Superior de Agronomia, Universidade de Lisboa, Tapada da Ajuda, 1349-017 Lisboa, Portugal

**Keywords:** *Daucus carota*, alternative oxidase, growth, chilling stress, *cis*-elements

## Abstract

Alternative oxidase (AOX) is a key enzyme of the alternative respiration, known to be involved in plant development and in response to various stresses. To verify the role of *DcAOX1* and *DcAOX2a* genes in carrot tap root growth and in response to cold stress, their expression was analyzed in two experiments: during root growth for 13 weeks and in response to a cold challenge trial of 7 days, in both cases using different carrot cultivars. Carrot root growth is initially characterized by an increase in length, followed by a strong increase in weight. *DcAOX2a* presented the highest expression levels during the initial stages of root growth for all cultivars, but *DcAOX1* showed no particular trend in expression. Cold stress had a negative impact on root growth, and generally up-regulated *DcAOX2a* with no consistent effect on *DcAOX1*. The identification of cis-acting regulatory elements (CAREs) located at the promoters of both genes showed putative sequences involved in cold stress responsiveness, as well as growth. However, *DcAOX2a* promoter presented more CAREs related to hormonal pathways, including abscisic acid and gibberellins synthesis, than *DcAOX1*. These results point to a dual role of *DcAOX2a* on carrot tap root secondary growth and cold stress response.

## 1. Introduction

The plant mitochondrial electron transport chain (ETC) is branched so that electrons in the ubiquinone pool can pass to oxygen via the usual cytochrome (cyt) pathway (involving Complex III, cyt c and cyt oxidase) or via alternative oxidase (AOX) [1]. Electron flow from ubiquinol to oxygen via the cyt pathway is coupled to proton translocation and hence contributes to the synthesis of ATP, whereas electron flow from ubiquinol to oxygen via AOX is not coupled to proton translocation, hence not contributing to ATP synthesis, with most of the energy dissipating as heat [2,3]. The presence of AOX provides the respiratory system with built-in flexibility regarding the degree of coupling between carbon metabolism pathways, electron transport chain activity, and ATP turnover [4].

AOX can play a role in response to stress and on the maintenance of cellular and mitochondrial homeostasis [5], and numerous studies have focused on the role of AOX under biotic and abiotic stressful growth conditions [6,7,8]. Low temperature is an adverse abiotic factor that strongly influences the growth, productivity, and quality of crops [9]. However, plants growing in temperate regions can become tolerant to low temperatures through a process termed cold acclimation [10,11]. The involvement of AOX on this physiological process has been reported in several plant species [8,12,13].

There are considerably less studies on the involvement of AOX in the response to biotic stress. However, it has been observed that the transcriptional responses to both abiotic and biotic stresses have a significant overlap [14,15]. In the case of pathogen attacks, for example, a salicylic acid-mediated reaction aiming to limit pathogen spread is observed, along with an increase in AOX expression, such as that which occurs in response to abiotic stress [16]. Besides its involvement in plant-pathogenic organisms’ interaction, *AOX* genes have been also associated with response upon beneficial microorganism interactions, both at host plant and symbiont microorganism sides [17,18].

In higher plants, AOX is nuclear encoded by a small multigene family, comprising one to six gene members distributed within two subfamilies, the *AOX1* and *AOX2*-subfamily [19,20,21]. The number of gene members and the pattern of its ramification within the two subfamilies is species-dependent [22,23,24,25,26]. The carrot (*Daucus carota* L.) *AOX* gene family is composed by three gene members with ramification at the *AOX2*-subfamily, *DcAOX2a,* and *DcAOX2b* [27,28]. Due to its involvement in plant response upon environmental constrains, several studies have been conducted to investigate the diversity on *AOX* genes sequences in view of further development of molecular and functional markers associated to plant plasticity [29,30,31,32,33,34]. This was successfully attained in olive with the identification of specific polymorphisms in *OeAOX2* associated with the capacity to easily develop adventitious roots [35] and, more recently, by the identification of a SNP variant in the single watermelon (*Citrullus lanatus*) *ClAOX* gene associated to cold stress tolerance [36].

The cultivated carrot is one of the most important vegetable plants in the world due to its high yield potential and use as fresh or processed product, with increased awareness of its health benefits associated with human consumption [37]. With an annual world production (carrots and turnips) of >44.7 million tons hectares [38], carrots rank among the top 10 vegetable crops in the world [37] with recognized agronomic interest. Additionally, carrots have been used as a model plant to study different biological systems (for example, studies focused on cellular totipotency and morphogenesis) [26,39].

*AOX* genes also showed differential expression in plant organs and tissues [28,40,41], and its involvement in plant developmental processes was shown during seed germination and seedlings development [20,42,43], vegetative growth, and reproductive performance [43,44,45,46]. Moreover, *AOX* genes were associated with cell reprogramming processes, in some cases related with physiological and morphological changes [47,48,49].

Carrot *AOX* genes were already found to be differentially transcribed in various systems and associated with cell reprogramming processes. This includes de novo growth from quiescent root phloem tissue [27,28] and somatic embryogenesis [39]. Also, *DcAOX* revealed an early transcription increase in response to chilling [27].

The involvement of different signaling pathways in AOX induction have been reported [50] and the role of the promoter region in driving that expression has been investigated at different levels [51,52,53,54]. The role of *cis*-acting regulatory elements (CAREs) located within the promoter sequence of *AOX* genes has been demonstrated by driving the expression of reporter genes using promoter deletion constructs [51,52]), by mutagenesis studies [53], and more recently by comparative analysis following in silico bioinformatics studies [54,55].

In the present study it was hypothesized that *AOX* genes (specifically *AOX1* and *AOX2a*) are involved in carrot secondary tap root growth and on plant response upon temperature challenge. For that, gene expression was measured during root development and also under cold stress, in several carrot cultivars. In an attempt to understand the differences on gene expression, an in silico analysis for identification of *cis*-regulatory elements was performed at the promoter region of both genes.

## 2. Results

### 2.1. Carrot Tap Root Development and Cold Challenge

**Figure 1:**
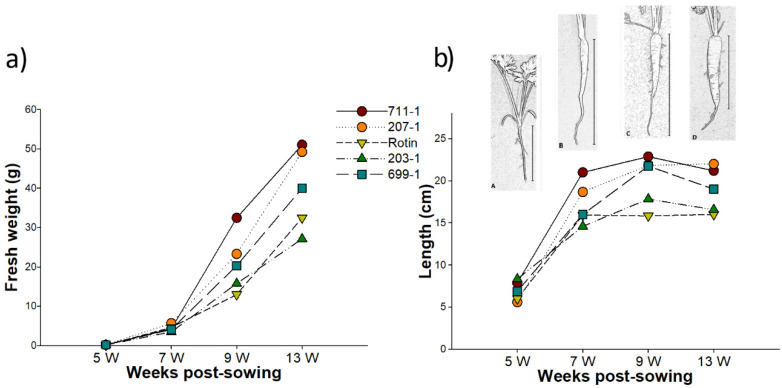
Measurements of carrot tap roots at 5, 7, 9, and 13 weeks post sowing. (**a**) Fresh weight (g) and (**b**) root length (cm) with a carrot scheme representing the general aspect of carrot tap roots at (A) 5, (B) 7, (C) 9, and (D) 13 weeks post sowing. The vertical bar in (b) indicates the part of the tap root used for length measurement.

Carrot tap root fresh weight (FW) and length measurements were taken from developing roots belonging to five different carrot cultivars (cv.) (711-1, 207-1, 203-1, 699-1 and ‘Rotin’) at 5, 7, 9, and 13 weeks post-sowing (wps), the same time points further considered for gene expression analysis. Within each cultivar, FW slightly increased from 5 to 7 wps, without significant differences being detected (*p* > 0.05), then followed by a marked increase until 13 wps, being significantly higher than at 5 wps (*p* < 0.05). FW mean value varied according to the cultivar, with cv. 711-1 and 207-1 reaching the highest and the 203-1 the lowest values at 13 wps (Figure 1a). Contrarily to FW, root length sharply increases at early development (Figure 1b). The part of the tap root used in length determination is indicated in Figure 1b (A, B, C, D). Between 5 wps and 7 wps a great increase occurred in all cultivars, with significant differences of at least *p* < 0.05 (Figure 1b, Table S1). Between 9 and 13 wps a decrease on root length mean value was observed in almost all cultivars (exception for 207-1), and significantly different in 203-1 (Figure 1b, Table S1). This resulted from the fact that at 13 wps only the clearly distinguishable storage tap root was considered to take the length value, i.e., the final part of the carrot tap root was not taken into account, as in some roots it was noticed that this part was broken during harvest due to its low thickness (see Figure 1b).

**Figure 2:**
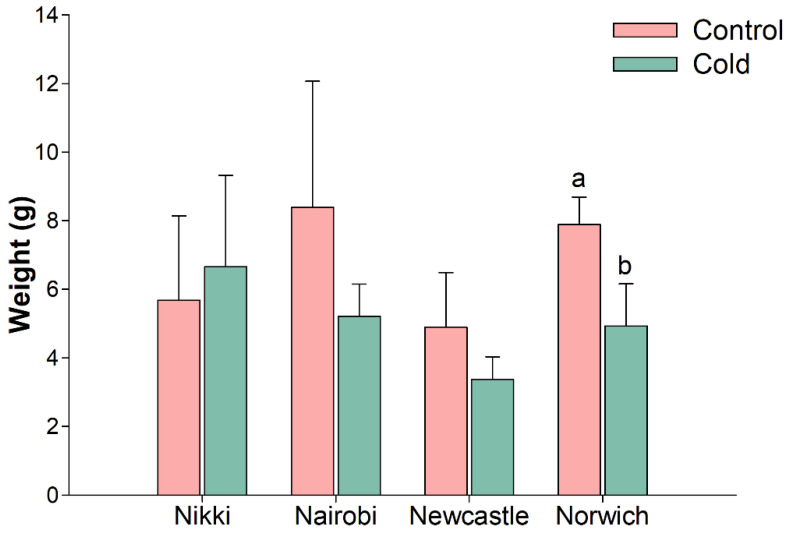
Root fresh weight seven days after initiation of cold challenge experiment in carrot cultivars ’Nairobi’, ‘Newcastle’, ‘Nikki’, and ‘Norwich’. Plants from control were always grown at 25 °C and samples from cold treatment were grown at 5 °C during seven days. Significant difference in cultivar ‘Norwich’ is indicated by different letters (*p* < 0.05). Carrot plants are shown in Figure S1.

For the cold challenge trial, four different carrot cultivars (‘Nairobi’, ‘Nikki’, ‘Newcastle’ and ‘Norwich’) were used at eight weeks post sowing. A 5 °C cold-stress in controlled growth chamber environment was applied during seven days, along with control plants (25 °C). Tap root samples were obtained from both cold-stressed and control plants at 4 h, 8 h, 24 h and seven days of cold exposure (hereinafter designated by T1, T2, T3, and T4), the same time points further considered for gene expression analysis. At T4, a general decrease in tap root FW was observed in 5 °C plants, which was significantly different in the cv. ‘Norwich’ (*p* < 0.05) (Figure 2).

### 2.2. DcAOX Expression during Carrot Root Development and Cold Challenge Trial

The transcription profiles of *DcAOX1* and *DcAOX2a* were analyzed by Reverse-Transcription quantitative PCR (RT-qPCR) in different cultivars of carrot during secondary tap root development until 13 wps, and after a cold challenge of 5 °C during seven days.

**Figure 3:**
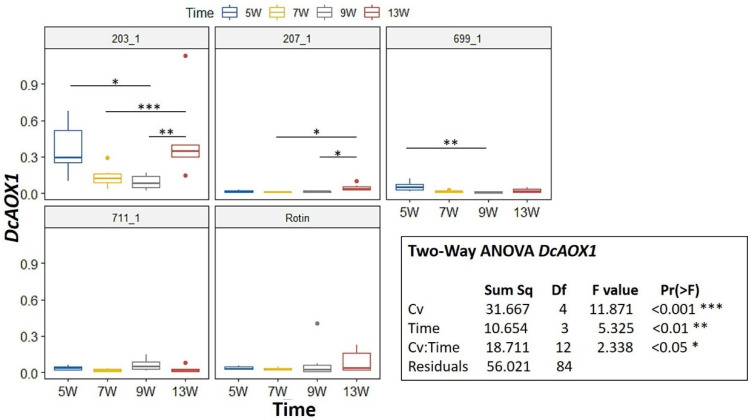
Expression box-plot and two-way ANOVA analysis for *DcAOX1* during carrot root secondary growth in the cultivars (Cv) 203-1, 207-1, 699-1, 711-1 and ‘Rotin’. Transcript levels were determined by RT-qPCR. For each time point, four to six biological replicates were considered per cultivar. Significant differences in gene expression between time points are indicated by * (*p* < 0.05), ** (*p* < 0.01) or *** (*p* < 0.001). Boxplots show the distributions (median, spread and outliers) of the gene expression values.

**Figure 4:**
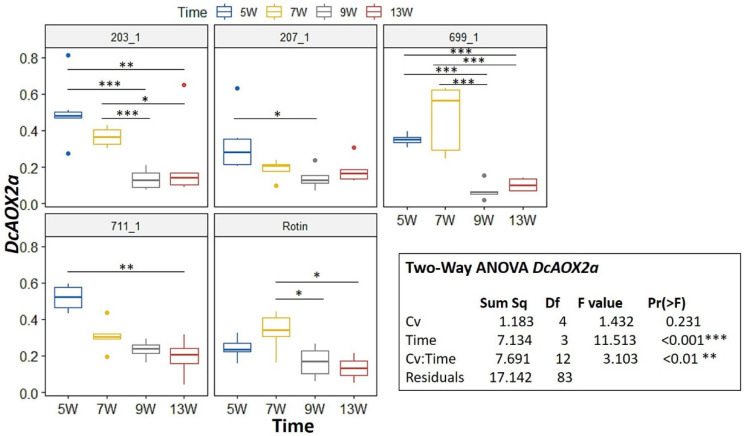
Expression box-plot and two-way ANOVA analysis for *DcAOX2a* during carrot root secondary growth in the cultivars (Cv) 203-1, 207-1, 699-1, 711-1, and ‘Rotin’. Transcript levels were determined by RT-qPCR. For each time point, four to six biological replicates were considered per cultivar. Significant differences in gene expression between time points are indicated by * (*p* < 0.05), ** (*p* < 0.01), or *** (*p* < 0.001). Boxplots show the distributions (median, spread and outliers) of the gene expression values.

During development, a Two-Way ANOVA revealed a significant interaction for *DcAOX1* between factors Cv*Time (*p* < 0.05) (Figure 3). Hence, we further pairwise compared the effects of time on every Cv using an emmeans test. For *DcAOX1*, the cultivar that showed more changes in gene expression was 203_1, being also the one showing the highest expression (Figure 3). For 711-1 and ‘Rotin’ there were no differences in expression between time points. Regarding *DcAOX2a*, there was also a significant interaction between Cv*Time (*p* < 0.01) (Figure 4). The expression patterns of 699-1 and ‘Rotin’ slightly differed from the rest of the cultivars, presenting the highest expression at 7 wps, followed by a marked decrease (Figure 4).

**Figure 5:**
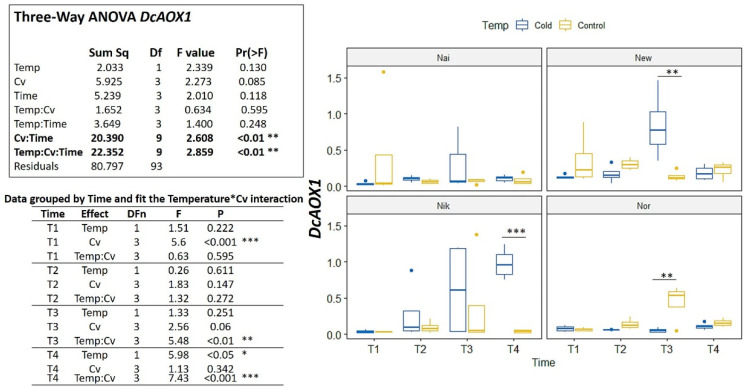
Expression box-plot and three-way ANOVA analysis for *DcAOX1* during cold challenge (T1: 4 h, T2: 8 h, T3: 24 h, and T4: 7 days) in carrot cultivars (Cv) ’Nairobi’ (Nai), ‘Newcastle’ (New) ‘Nikki’ (Nik), and ‘Norwich’ (Nor). Transcript levels were determined by RT-qPCR. For each time point, four biological replicates were considered per temperature. Significant differences between temperatures for the same time point are indicated by * (*p* < 0.05), ** (*p* < 0.01) or *** (*p* < 0.001). Boxplots show the distributions (median, spread and outliers) of the gene expression values.

**Figure 6:**
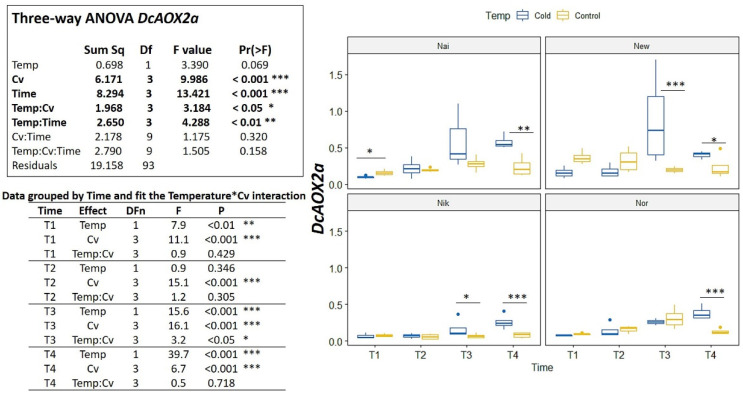
Expression box-plot and three-way ANOVA analysis for *DcAOX2a* during cold challenge (T1: 4 h, T2: 8 h, T3: 24 h and T4: 7 days), in carrot cultivars (Cv) ’Nairobi’ (Nai), ‘Newcastle’ (New) ‘Nikki’ (Nik), and ‘Norwich’ (Nor) eight weeks after sowing. Transcript levels were determined by RT-qPCR. For each time point, 10–12 biological replicates were considered per temperature. Significant differences between temperatures for the same time point are indicated by * (*p* < 0.05), ** (*p* < 0.01) or *** (*p* < 0.001). Boxplots show the distributions (median, spread and outliers) of the gene expression values.

In the cold challenge trial, significant interactions were detected among Cv*Time and Temp*Cv*Time (*p* < 0.01) (Figure 5). For *DcAOX1*, the cultivars showed no common trend of expression for either time or temperature (Figure 5). On the contrary, *DcAOX2a* showed a general increase in expression on the cold treatment for all cultivars (variable Time *p* < 0.001) (Figure 6). In fact, when grouping data by time (Figure 6) it is clear to see that at T4, the *DcAOX2a* expression in the 5 °C treatment was much higher than in the control plants, which remained stable throughout the entire trial (Figure 6).

### 2.3. Identification of cis-Regulatory Elements

**Figure 7:**
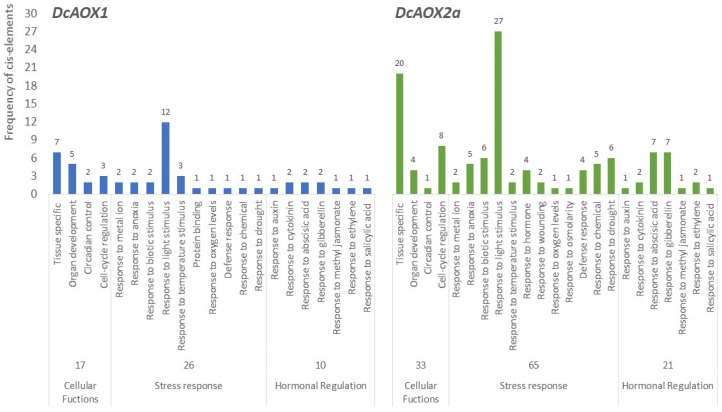
Frequency of *cis*-regulatory motifs identified in 2.0 Kb promoter region of both *DcAOX* genes (*DcAOX1* and *DcAOX2a*) using New PLACE and PlantCARE software’s.

The region up to 2.0 kbp upstream from the translation start site of both *DcAOX1* and *DcAOX2a* was scanned using PlantCare and NewPlace (databases of plant *cis*-acting regulatory DNA elements) for the identification of *cis*-acting regulatory elements putatively involved in plant growth and cold stress response. The analysis revealed a total of 52 putative CAREs in *DcAOX1* while in *DcAOX2* a total of 117 CAREs were identified. Accordingly with its putative functionality, CAREs were grouped into different categories (Figure 7): (i) related with cellular functions, (ii) responsive to stress conditions, and (iii) involved in hormonal regulation. In both genes, CAREs directly involved in plant stress response were the most frequent (*DcAOX1* exhibited 25 and *DcAOX2a* 63). Nevertheless, the existence of crosstalk networks among phytohormones and target genes that enable plants to efficiently react upon stress, here grouped in the functional category of hormonal regulation, cannot be neglected. Supplementary Tables S2 and S3 list all CAREs identified by the in silico analysis.

**Figure 8:**
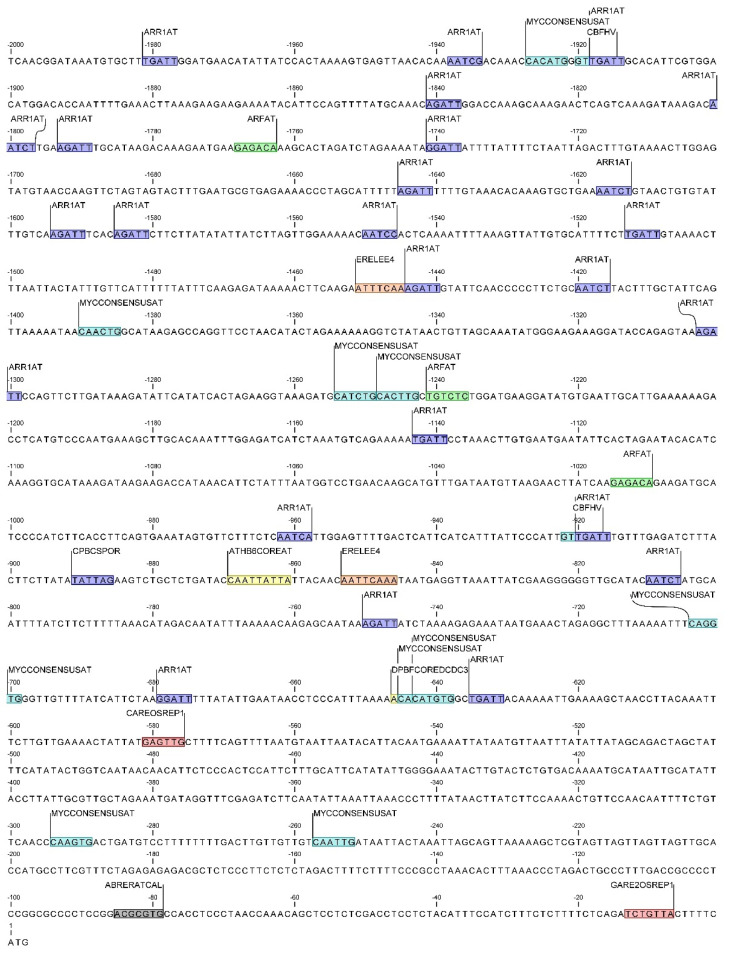
Location of *cis*-regulatory elements within the *DcAOX1* promoter, involved in cold-stress response (in light blue) in response to plant hormones: auxin (green), cytokinin (dark blue), abscisic acid (yellow), gibberellin (red), ethylene (orange), and calcium (grey).

**Figure 9:**
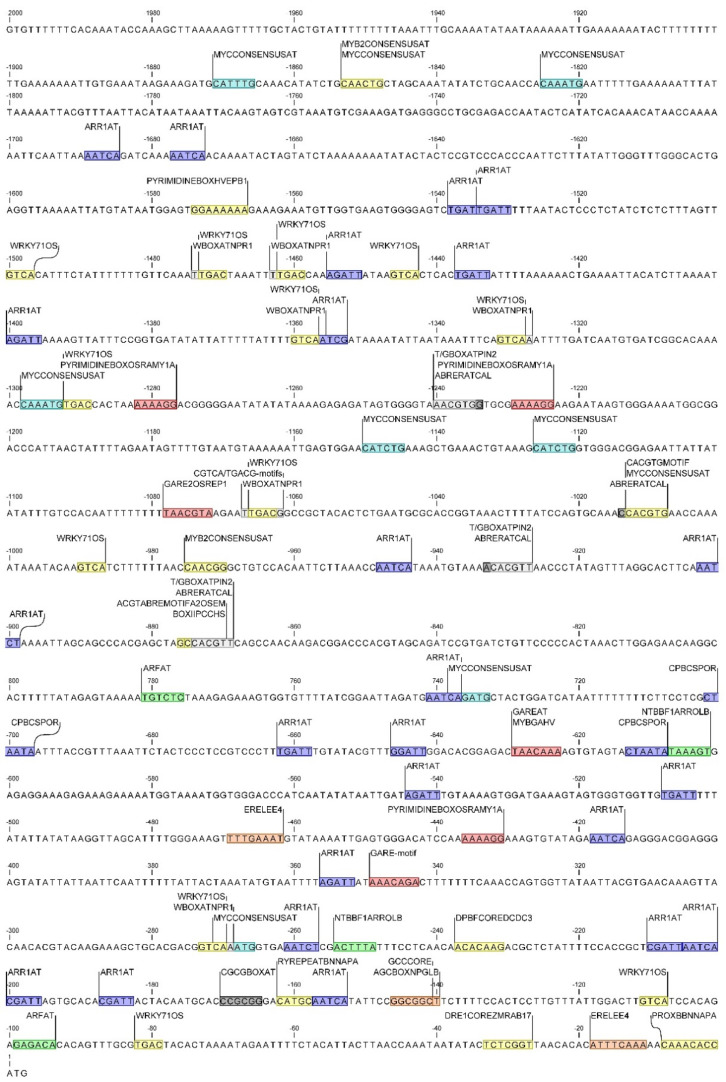
Location of *cis*-regulatory elements within the *DcAOX2a* promoter’s, involved in cold-stress response (in light blue), in response to plant hormones: auxin (green), cytokinin (dark blue), abscisic acid (yellow), gibberellin (red), ethylene (orange), and calcium (grey).

Within the temperature responsive CAREs, putative sequences involved in cold stress responsiveness were identified in both *DcAOX* members (Figure 8 and Figure 9). The elements identified using the NewPlace databases included the CBFHV, only identified in the *DcAOX1*, and the MYCCONSENSUSAT identified in both genes (for details related to position see Figure 8 and 9).

Additionally, several CAREs involved in calcium responsiveness were identified within the category of stress-responsive *cis*-regulatory elements. ABRERACAL involved in calcium and abscisic acid (ABA) response, and CGCGBOXAT, a calmodulin-binding/CGCG box, was identified in a single region of both promoters.

*cis*-Acting regulatory elements involved in hormonal regulation that could eventually be involved in stress responses were identified in the promoter regions of both genes. In *DcAOX1* promoter several of these motifs were identified: ABA-responsive elements DPBFCOREDCDC3 and ATHB6COREAT; the ethylene-responsive element ERELEE4 (ERE); the auxin-responsive element ARFAT, the gibberellin (GA)-responsive elements CAREOSREP1 and GARE2OSREP1; and the cytokinin responsive *cis*-elements ARR1AT and CPBCSPOR (see locations at Figure 8).

The promoter region of *DcAOX2a* presents a higher diversity of CAREs involved in hormonal regulation than *DcAOX1* (locations can be seen in Figure 9). Besides three CAREs sites common to both *DcAOX* genes (DPBFCOREDCDC3, ERELEE4, and ARFAT), a high number of gene-specific CAREs were found in *DcAOX2a*. The ABA-responsive elements were: ACGTABREMOTIFA2OSEM (also identified at PlantCARE as ABRE), DRE1COREZMRAB17, PROXBBNNAPA, RYREPEATBNNAPA, MYB2CONSENSUSAT, BOXIIPCCHS, CACGTGMOTIF, WRKY71OS, and PYRIMIDINEBOXHVEPB1, these last two also being involved in response to gibberellin. In addition to those two CAREs, five other motifs specific of plant cell response upon GA were found, which includes GAREAT, GARE-motif, GARE2OSREP1, MYBGAHV, and PYRIMIDINEBOXOSRAMY1A. *cis*-Regulatory elements involved in ethylene-response recognized in *DcAOX2a* promoter sequence were the GCCCORE and the AGCBOXNPGLB; the auxin-responsive element NTBBF1ARROLB; and the cytokinin responsive *cis*-elements ARR1AT and CPBCSPOR were also found. *cis*-Regulatory element T/GBOXATPIN2 involved in salicylic acid (SA), WBOXATNPR1 and CGTCA/TGACG-motifs involved in jasmonate (JA)/metyl jasmonate (MeJa) signaling pathways were identified.

In *DcAOX1* several CAREs putatively involved in the regulation of genes linked to root growth were identified: the E2FAT, E2FANTRNR, and E2F1OSPCNA, involved in cell-cycle, and WUSATAg and XYLAT, associated to root apical meristem and secondary xylem development, respectively. In *DcAOX2a* it was also identified the WUSATAg, and three additional gene specific CAREs. Those comprise the CCGTCC-box and dOCT, which is related to meristem specific activation. The third one corresponds to the MYBCOREATCYCB1 involved in cell-cycle.

## 3. Discussion

Root meristems located in the cambium ring are the main tissue responsible for secondary growth in carrot tap roots [34], starting its development around four to seven weeks after sowing [56]. Located between the primary xylem and the phloem, the cambium ring produces phloem tissue on the outside and xylem tissue on the inside [56]. Therefore, it is very likely that at the initial time point presented in this work (5 wps), the secondary growth of carrot tap roots was at the very beginning, since a pronounced increase in root biomass was only observed after 7 wps. On the contrary, during initial stages (between 5 and 7 wps) the root length sharply increased, as observed by Palussek and Neumann [57], which referred that root length is determined previously to the root secondary growth.

AOX has been shown to be especially active in meristematic tissues [58] and several studies have indicated a connection between AOX activity and plant growth and development [4,59]. For instance, in soybean, an antisense knockdown of *GmAOX2b* was shown to compromise both vegetative growth and seed yield under typical greenhouse growth conditions [46]. In our study, and unlike *DcAOX1*, *DcAOX2a* followed a concrete trend during carrot storage root growth, presenting the highest expression values between 5 and 7 wps (depending on the cultivar), just before the initiation of the secondary growth. During that period no significant increase of weight was observed, while root length greatly increased in all the studied cultivars. At a later stage, when *DcAOX2a* expression was reduced (at 9 and 13 wps), and when higher cell division rates are likely to take place in the meristem (reflected as secondary growth), the increment on root length stopped.

The available reports demonstrate that *AOX* expression patterns are not constant across species. In soybean, the relative abundance of soybean *GmAOX2* decreased during seedling development, whereas the transcript abundance of other *GmAOX* genes increased [43]. Saisho et al. [42] observed that *AtAOX2* expression in *Arabidopsis* was high in dry seeds and subsequently decreased during early germination, whereas *AtAOX1a* was less abundant at the beginning of the process and only increased in a later stage. A putative role for *AOX* on post-germinative development of *Hypericum perforatum* seedlings was also suggested, with *HpAOX1* expression showing a marked increase during that process [20]. On the contrary, *HpAOX2* transcripts demonstrated a greater stability. It seems therefore that the involvement of *AOX* genes on growth and development is highly species-specific, and not only related with the gene sub-family (*AOX1* or *AOX2*) but also with the specific function of each gene within the sub-family.

As sessile organisms, plants have developed highly sophisticated and intricate defence mechanisms allowing them to overcome freezing constraints, which involves the mitochondria as a physical platform for networks, signal perception and signal canalization ([33] and reference therein). The AOX, a protein located at the inner mitochondrial membrane, with a key role on alternative respiratory pathway, takes part on plant response and adaptation upon cold stress, acting on signal perception and subsequent intraorganellar cross-talk signalling pathway (retrograde signalling) by mediating the level of energetic molecules (NAD(P)H and ATP/ADP) and reactive oxygen species (ROS) [60]. The involvement of AOX on plant acclimation to cold stress could be directly linked to the important role in decreasing the mitochondrial ROS level by reducing oxygen to water without conservation of energy in the form of ATP [61]. In protoplasts of *Pisum sativum* subjected to sub-optimal temperatures, it was shown that AOX pathway optimizes photosynthesis by regulating ROS, malate valve and antioxidative systems [12]. Studies conducted by site-directed mutagenesis in *O. sativa* (*OsAOX1a*), demonstrated the link with a quantitative trait locus for thermo tolerance [62], and recently Ding et al. [36] demonstrated by ectopic expression of *ClAOX* alleles differing by a SNP mutation, a cold tolerance increase in *Arabidopsis* aox1a knock-out mutant.

AOX has been reported to be responsible for the development of cold resistance in winter wheat seedlings and in response to cold stress in tobacco [63,64]. Also, Fiorani et al. [44] observed a reduced leaf area and rosette size through the antisense suppression of *AtAOX1a* in *Arabidopsis* plants grown for 21 days at 12 °C. Such differences decreased as the plants approached flowering, suggesting that *AtAOX1a* played a role in the acclimation of shoot growth to low temperature during early vegetative development. In the present work, the results obtained in the cold challenge trial point to a greater involvement of *DcAOX2a* on the process of cold response than *DcAOX1*. It should be taken in consideration that AOX isoforms are not redundant and cannot compensate for each other under stress or adverse growth conditions, as revealed by studies on various transgenic plants (reviewed by [1]).

The specificities of *AOX* gene family amongst plant species include differences in gene structure and the presence of regulatory elements within the different components of the gene. Within the diversity of elements with a known role in driving gene expression, the *cis*-acting regulatory elements, 5–20 bp in size and located within the promoter sequence, are recognized as important factors in regulating gene expression upon specific conditions [65]. Low temperature stress is one of the main abiotic stress factors causing strong impact on plant growth and development. In carrot *DcAOX1* and *DcAOX2a* promoter regions there were found C-repeat binding factors (CBFs), which are members of the AP2/ERF transcription factor family, playing a fundamental role in regulating cold-responsive genes and cold acclimation [66]. Following exposure to low temperature stress, genes encoding CBF proteins are rapidly and transiently induced via an ABA–independent pathway, and their products activate the CBF regulon, cold-stress related genes involved in plant tolerance and acclimation [67]. The role of CBFs upon temperature stress has been described in a diversity of plant species, such as barley [68], rice [69], *Arabidopsis* [70] and wheat [71]. The MYCCONSENSUSAT, a binding site essential for CBF3 to respond to cold stress in maize [72], *Arabidopsis* [73] and cotton [74], was in silico identified in the promoter region of both carrot *AOX* genes. If considering the CAREs directly linked to cold-stress response, the *DcAOX1* would be selected as putatively more responsive due to the presence of an additional CBF binding site, the CBFHV, previously described in barley [75] and cotton [74], as also involved in cold-stress response. However, expression analysis of both carrot *AOX* genes upon cold treatment revealed a higher responsiveness of *DcAOX2a*, which cannot be explained solely based on the CAREs above described. It is interesting to note that a higher diversity and frequency of CAREs belonging to each category (cellular function, stress response and hormonal regulation) was identified at the *DcAOX2a* gene promoter (Figure 8 and Figure 9). Considering that the number of binding sites is involved in controlling gene expression [76], the higher number of CAREs identified in *DcAOX2a* can explain the greater responsiveness of this gene to a wider range of environmental stress factors and to morphogenic/developmental processes.

CAREs responsive to plant hormones are also connected to plant response upon cold stress. In comparison to *DcAOX2a*, the *DcAOX1* lost almost half the CAREs associated to hormonal response (mainly related to ABA and GA), which suggest a relevant link between *DcAOX2a* function and the metabolism of some hormones. Under cold stress, ABA, GA, auxins, citokinins, ethylene, brassinosteroids (BRs), salicylic, and jasmonic acid (JA) interact in a complex crosstalk acting as central regulators controlling plant response (see review in [77] and references therein). The role of ABA as signaling molecule acting as mediator on regulation plant plasticity upon diverse environmental constrains has been highlighted by different reports (see review [78] and references therein, [79]). Wang et al. [60] recently described the link between ABA and SA signalling pathways, and the crosstalk between both phytohormones and H_2_O_2_ on mediating wheat plant tolerance to cold stress. Within the different ROS molecules that can exist in the cells, the H_2_O_2_ has been described as the ROS messenger responsible for long-distance transport in cells acting as second messenger in phytohormone signalling, responsible for the induction of downstream target genes [60,80]. Evidence have shown that ABA-induced H_2_O_2_ accumulation may be involved in transcription activation of diverse antioxidative genes, contributing for cellular homeostasis and consequently plant acclimatization [60]. Promoter sequence of *DcAOX2a*, exhibiting high number of CAREs related to ABA responsiveness, could represent an important indication about the involvement of that gene on carrot plant response upon cold stress mediated by specific phytohormone signaling pathways. In carrot, most reports about ABA are related to somatic embryogenesis (see review [81]), and there is no information regarding its involvement in carrot plasticity to abiotic stresses. Nevertheless, early studies developed in different plant species reported ABA accumulation in response to cold [82,83]. Knowing that the level of ABA in plants usually increases during abiotic stress conditions, and that elevated ABA levels can enhance plant adaptation to various stresses [84], the high diversity/frequency of ABA *cis*-regulatory elements located at *DcAOX2a* promoter could explain the higher responsiveness of this gene to cold-stress, controlled by endogenous levels of accumulated ABA. Furthermore, the identification in *DcAOX2a* promoter of seven CAREs involved in GA-responsiveness suggests a regulation of plant cold response through GA signalling pathway. Gibberellins have been linked to stress tolerance at both levels, metabolic [85] and signalling [86], playing critical roles in conferring plants the ability to adapt their growth to changing environmental conditions. Moreover, a crosstalk between GA and CBFs has been demonstrated in *Arabidospsis*, tobacco and cotton [85,87].

Besides the involvement in plant response to environmental constrains, several hormonal pathways, including auxin, cytokinin, ethylene, GA, BRs, JA, ABA, and strigolactone converge on the regulation of root growth (see review at [88]). The high number of hormone-responsive CAREs at the promoter region of *DcAOX2a* can also contribute to the possible higher involvement of this gene in carrot root growth when comparing to *DcAOX1*. In addition, several CAREs involved in plant growth and development in other plant species were also identified in carrot. The motif WUSATAg, identified in both carrot genes, was identified in the promoter of a rice WUSCHEL-type homeobox gene, expressed in the root apical meristem [89]. In addition, XYLAT, identified in *DcAOX1* was reported in *A. thaliana* and *Leucaena leucocephala* with a relevant role to the secondary xylem cell differentiation [90,91].

## 4. Materials and Methods

### 4.1. Experimental Setup

To test the hypothesis that *AOX* genes are involved in carrot root growth and cold stress tolerance, two different experimental trials were performed.

For the secondary root growth experiment, five different carrot cultivars were used: 711-1, 207-1, 203-1, and 699-1 (cultivated carrot breeding stocks developed by the USDA carrot breeding program, gently provided by Prof. Phillip Simon, Wisconsin University, USA), and the cv. ‘Rotin’. Seeds of each cultivar were sown in pots containing commercial substrate (SIRO Plant, Leal & Soares, S.A., Portugal) and maintained under greenhouse conditions for 13 weeks. Three pots with 10 plants per pot were considered per cultivar. Harvest was performed randomly considering four to six biological replicates (single plants) collected at different time points: 5, 7, 9 and 13 wps. Fresh weight (g) and root length (cm) of each tap root were annotated. Complete roots (for samples collected at 5 and 7 wps) or pieces from the upper third-part of the tap root (for samples collected at 9 and 13 wps) were snap frozen in liquid nitrogen and stored at −80 °C until further processing.

Four different carrot cultivars were used for the cold challenge trial: ‘Nairobi’, ‘Nikki’, ‘Newcastle’, and ‘Norwich’ (gently provided by Bejo Seed Company, The Netherlands). Seeds of each cultivar were sown in 6 pots with 10 plants per pot containing commercial substrate (SIRO Plant, Leal and Soares, S.A., Portugal). Plants were maintained in a growth chamber Model Fitoclima D1200PLH from Aralab^®^ (Portugal) for eight weeks, at constant temperature of 25 °C, 80% humidity and 16 h photoperiod, 95–100 μmol m^−2^s^−1^ light intensity provided by Osram L fluorescent tubes 36W/840. Following that, half of the plants were exposed to 5 °C for 7 days, and half were maintained under the initial conditions of 25 °C (control). Root samples consisting of roots pieces from the upper third-part of the tap root were taken from both cold stressed plants and control plants at different timepoints after cold exposure: 4 h, 8 h, 24 h, and seven days (T1, T2, T3 and T4, respectively). Four plants (biological replicates) were randomly collected at each time point for each cultivar and temperature condition. Samples were snap frozen in liquid nitrogen and stored at −80 °C until further processing.

### 4.2. RNA Extraction and cDNA Synthesis

Total RNA was isolated from carrot roots using the RNeasy Plant Mini Kit (Qiagen, Hilden, Germany), with on-column digestion of DNA with the RNase-Free DNase Set (Qiagen, Hilden, Germany), according to manufacturer’s protocol. The quantification of RNA and evaluation of its quality were determined in a NanoDrop-2000C spectrophotometer (Thermo Scientific, Wilmington, DE, USA). The integrity was evaluated by denaturing gel electrophoresis and visualised using a Gene Flash Bio Imaging system (Syngene, Cambridge, UK) after staining in an EtBr solution (2 ng·mL^−1^).

DNase-treated total RNA (1 µg) was reverse transcribed with random decamer primers, using the RETROscript^®^ kit (Ambion, Austin, TX, USA) according to manufacturer’s instructions.

### 4.3. Gene Expression Analysis by RT-qPCR

Gene-specific primers were designed to detect the transcripts of the most responsive carrot *AOX* genes (*DcAOX1* and *DcAOX2a*) [27,28]. The Primer Express Software (Applied Biosystems, Foster City, CA, USA) was used considering the default parameters. Carrot *EF-1A, GAPDH*, and the Ribosomal 5.8S (*5.8S rRNA*) genes were used as candidate reference genes [92]. Primer sequences and amplicon sizes are shown in Table S4.

Quantification of gene expression was performed by RT-qPCR with SYBR Green q-PCR Master Mix (Fermentas, Ontario, Canada) on a 7500 Real Time PCR System (Applied Biosystems, Foster City, CA, USA). 18 μL reaction volume containing 5 µL of first-strand cDNA (previously diluted 1:10) and 560 nM of each specific primer was used for expression analysis. RT-qPCR was conducted for 40 cycles, each consisting in 15 s at 95 °C followed by 1 min at 60 °C. To analyse the dissociation curve profiles, an additional step at 95 °C during 15 s was added, followed by a constant increase of temperature between 60 °C and 95 °C. All samples were run in triplicate. To assess the possibility of contaminations and primer dimmers a minus reverse transcriptase and no-template controls (NTCs) were included for the five pair of primers. Amplification specificity of each primers pair was confirmed by performing a melting curve analysis after each PCR run. Amplification efficiency (E) (Table S4) was calculated using the formula E = (10^(−1/slope)^ − 1) × 100, where the slope of the standard curve was determined by the Applied Biosystems (AB) software. Standard curve was performed using undiluted pool of all cDNA samples and three four-fold serial dilutions (1:1–1:125).

Cq values were acquired for each sample with the Applied Biosystems 7500 software (Applied Biosystems, Foster City, CA, USA). Evaluation of expression stability for the candidate reference genes (*EF-1A*, *GAPDH* and *rRNA5.8S*) was carried out using the statistical application *geNorm* [93], which selected *EF-1A* and *GAPDH* as the most stable genes with no need to include the third gene for normalization. Expression of target genes was evaluated by relative quantification using the geometric normalisation factors obtained from *geNorm*.

### 4.4. Statistical Analysis

Differences in carrot length and weight during the growth experimental trial were analysed by One-way analysis of variance (ANOVA) using the R package ’rstatix’. For the cold experiment, root fresh weight following seven days of cold stress was compared with control plants using the Student’s *t*-test. Significance levels were set at *p* < 0.05.

The expression of *DcAOX1* and *DcAOX2a* genes were analysed by Two-Way and Three-Way ANOVA for the root development and cold stress trials, respectively, with Bonferroni adjustment, using the R packages ‘tidyverse’, ‘ggpubr’ and ‘rstatix’. Expression data were log transformed to meet the normality and homogeneity of variances’ requirements. Pairwise comparisons were carried out using emmeans test, and a significant *p*-value < 0.05 was considered.

### 4.5. Analysis of Promoter Sequences for Identification of Cis-Responsive Elements Associated with Root Development and Cold Stress Response

To screen for the presence of *cis*-regulatory elements located at the promoter region that would be related with differential regulation of *DcAOX1 and DcAOX2a* gene expression during root development and upon cold stress, a region comprising 2.0 Kb upstream the translation start site of differentially expressed *AOX* members was considered for analysis. Promoter sequences were retrieved from the *D. carota* genome deposited at the PLAZA V4 databases (https://bioinformatics.psb.ugent.be/plaza/versions/plaza_v4_dicots/gene_families/view/HOM04D001520) [accessed on 10 January 2018]. Accessions DCAR_028361 and DCAR_021859 were used to retrieve the complete full length genomic sequence including upstream region in which the promoters of *DcAOX1* and *DcAOX2* genes are included. The freely available New PLACE tool—A Database of Plant *Cis*-acting Regulatory DNA elements (https://www.dna.affrc.go.jp/PLACE/?action=newplace) [accessed on 17 January 2018] [94] and PlantCARE (http://bioinformatics.psb.ugent.be/webtools/plantcare/html/ [accessed on 16 January 2018] [95] were used to in silico identify putative *cis*-regulatory elements. Both, New PLACE and PlantCARE, are databases of plant *cis*-acting regulatory DNA elements which have been collected from previous published reports.

The positions of the identified *cis*-elements were mapped at the promoter sequence using CLC Main Workbench 7.5.1 software (ClCbio, Aarhus N, Denmark).

## 5. Conclusions

Our work revealed *DcAOX2a* with the highest expression during the initial stages of root growth for all carrot cultivars, contrarily to *DcAOX1* that showed no specific trend in expression. Similar results were observed in carrot plants submitted to cold stress with *DcAOX2a* generally up-regulated, and no consistent changes on *DcAOX1*. Cold stress responsiveness as well as growth related CAREs were identified in both *DcAOX* genes. However, *DcAOX2a* promoter sequence presented more CAREs related to hormonal pathways, giving indications about its putative role on both, carrot tap root secondary growth and cold stress response.

In view of these results, *DcAOX2a* appears as a promising target for molecular marker development focused on selection of highly resilient carrot genotypes to be used in breeding programs.

## Data Availability

Data is contained within the article and Supplementary Materials.

## Supplementary Materials

The following are available online at https://www.mdpi.com/article/10.3390/plants10112369/s1. Figure S1: Carrot plants from cultivars ’Nairobi’, ‘Newcastle’, ‘Nikki’, and ‘Norwich’ seven days after initiation of cold challenge experiment. Plants from control were always grown at 25 ˚C and samples from cold treatment were grown at 5 °C during seven days; Table S1: Carrot root fresh weight and length at each time point; Table S2: Category wise list of *cis*-elements extracted from 2 Kbp upstream region of *DcAOX1* using PlantCare and PLACE.; Table S3: Category wise list of *cis*-elements extracted from 2 Kbp upstream region of *DcAOX2a* using PlantCare and PLACE; Table S4: Primers used in RT-qPCR. AS corresponds to amplicon size and E corresponds to primer efficiency.
