# Supplementary material for: Carrot AOX2a Transcript Profile Responds to Growth and Chilling Exposure
Source: Plants (Basel). 2021 Nov 3;10(11):2369. doi: 10.3390/plants10112369 (PMC8625938; doi:10.3390/plants10112369)
Supplement: Supplementary file 1 [file plants-10-02369-s001.zip › Table S1.pdf]

Table S1. Carrot root fresh weight and length at each time point

	203-1		207-1		´Rotin´		699-1		711-1	
	**Weight**	**Length**	**Weight**	**Length**	**Weight**	**Length**	**Weight**	**Length**	**Weight**	**Length**
**Time**	Average (g) ± SD	Average (cm) ± SD	Average (g) ± SD	Average (cm) ± SD	Average (g) ± SD	Average (cm) ± SD	Average (g) ± SD	Average (cm) ± SD	Average (g) ± SD	Average (cm) ± SD
**5W**	0.15^{c,d} ± 0.07	8.3^{b} ± 2.2	0.16^{c,d} ± 0.1	5.6^{b} ± 2.9	0.1^{c,d} ± 0.03	6^{b} ± 0.8	0.16^{b} ± 0.09	6.9^{b} ± 1.8	0.17^{b} ± 0.1	7.9^{b} ± 1.3
**7W**	3.4^{b,c} ± 1.5	14.6^{a} ± 1.9	5.7^{b,c} ± 3.6	18.7^{a} ± 2.7	4.6^{b,c} ± 2.3	15.9^{a} ± 3.3	4.1^{a,b} ± 2.9	16^{a} ± 5.5	4.2^{a,b} ± 1.5	21^{a,b} ± 2
**9W**	15.8^{a,b} ± 6.5	17.8^{a} ± 1.8	23.3^{a,b} ± 7.3	21.8^{a} ± 1.6	12.9^{a,b} ± 4.3	15.8^{a} ± 2.9	20.3^{a} ± 4.7	21.8^{a} ± 1.3	32.5^{a,b} ± 22.9	22.9^{a} ± 3.9
**13W**	27.1^{a} ± 10.9	16.6^{a} ± 2.6	49.2^{a} ± 13.1	22^{a} ± 2.8	32.5^{a} ± 13.4	16^{a} ± 3.1	39.9^{a} ± 24.9	19^{a} ± 4.7	51.0^{a} ± 22.4	21.2^{a} ± 3.6

Different superscript letters (a, b, c, d) indicate significant differences of at least $P < 0.05$ between time points, for weight or length, within each cultivar.
