# Supplementary material for: Carrot AOX2a Transcript Profile Responds to Growth and Chilling Exposure
Source: Plants (Basel). 2021 Nov 3;10(11):2369. doi: 10.3390/plants10112369 (PMC8625938; doi:10.3390/plants10112369)
Supplement: Supplementary file 1 [file plants-10-02369-s001.zip › Table S2.pdf]

Table S2 Category wise list of *cis*-elements extracted from 2 Kbp upstream region of *DcAOX1* using PlantCare and PLACE.

Categories based on functions	Extracted *Cis*-element (Tools used)		Sequence	Specific functions of *cis*-elements
	NewPlace	PlantCARE		
**Cellular Functions**	BOXIINTPATPB	-	ATAGAA	Organelle specific (plastid) gene expression
	CAATBOX1	-	CAAT	Tissue specific gene expression
	CACTFTPPCA1	-	TACT/CACT	Mesophyll-specific gene expression
	CEREGLUBOX2PSLEGA	-	TGAAAACT	Tissue specific legumin gene
	CCAATBOX1	-	CCAAT	Flowering regulation
	CCA1ATLHCB1	-	AAAAATCT	Phytochrome regulation
	CIACADIANLELHC	circadian	CAANNNNATC	Circadian control
	DOFCOREZM	-	AAAG	Sperm-specific expression during gametophyte development
	E2FAT	-	TTTCCCGCC	Cell cycle-regulated expression
	E2FANTRNR	-	TTTCCCGC	Cell cycle progression (G1/S transition)
	E2F1OSPCNA	-	GCGGGAAA	Transcriptional activation in dividing cells and tissues
	-	GCN4_motif	TGAGTCA	Endosperm specific gene expression
	-	HD-Zip 2	CAATCATTG	Control of leaf morphology development
	POLLEN1LELAT52	-	AGAAA	Anther and pollen development
	UP2ATMSD	-	AAACCCTA	Initiation of axillary bud outgrowth
	WUSATAg	-	TTAATGG	Regulation of stem cell population maintenance in shoots and floral meristems; regulation of genes expressed in the central cells of quiescent centers in the root apical meristem
	XYLAT	-	ACAAAGAA	Secondary xylem development
**Stress Response**	ABRERATCAL	-	CACGCGT	Calcium responsiveness
	ANAERO1CONSENSUS	-	AAACAAA	Anaerobically induced
	BIHD1OS	-	TGTCA	Disease resistance responses
	BOXCPSAS1	-	CTCCCAC	Light responsiveness
	-	Box 1	TTTCAAA<br>TGATAATGT<br>AAGATAAGA	Light responsiveness
	-	Box 4	ATTAAT	Light responsive
	-	CATT-motif	GCATTC	Light responsive
	CBFHV	-	ATCGAC	Cold responsive (freezing tolerance)
	CCAATBOX1	-	CCAAT	Heat shot enhanced expression (HSE)
	CGCGBOXAT	-	ACGCGT	Calmodulin-binding/CGCG box
	CURECORECR		GTAC	Copper- and oxygen-responsive

	EBOXBNNAPA	-	CANNTG	Regulates defense-related gene
	EECCRCAH1		GATTTTC<br>GAATTTC<br>GAATTGC<br>GAGTTGC	CO2-responsive transcriptional activation
	ELRECOREPCRP1	Box-W1	TTGACC	Pathogen- and wound-induced signaling
	GATABOX		GATA	Light regulated, and tissue specific expression
	-	GT1-motif	GGTTAA	Light responsive
	-	LAMP-element	CTTTATCA	Light responsive
	-	MBS	TAACTG<br>CGGTCA<br>CAACTG	Drought responsiveness
	MYCCONSENSUSAT	-	CANNTG	Cold responsive (freezing tolerance)
	SORLIP1AT	-	GCCAC	Light responsive on root -specific genes
	-	Sp1	CCGCCC	Light responsive
		TCT-motif	TCTTAC	Light responsive
		TCCC-motif	TCTCCCT	Light responsive
	-10PEHVPSBD	-	TATTCT	Light responsive
**Hormonal Regulation**	ABRERATCAL	-	MACGYGB	ABRE-related sequence as Ca2+-responsive
	ARFAT	-	TGTCTC	Auxin-responsive
	ARR1AT	-	TGATT	Cytokinin-regulated
	ATHB6COREAT	-	CAATTATTA	Abscisic acid (ABA) regulated
	CAREOSREP1	-	CAACTC	Gibberellin (GA)-responsive
	-	CGTCA-motif	CGTCA	Methyl jasmonate (MeJa) responsive
	CPBCSPOR		TATTAG	Cytokinin-responsive
	DPBFCOREDCDC3	-	ACACATG	ABA-responsive and embryo-specification elements
	ERELEE4	ERE	ATTTCAAA<br>AATTCAAA	Ethylene responsive
	GARE1OSREP1	-	TAACAGA	GA-responsive
	-	TCA-element	CCATCTTTTT<br>CAGAAAAGGA	Salicylic acid responsive
