# Supplementary material for: Carrot AOX2a Transcript Profile Responds to Growth and Chilling Exposure
Source: Plants (Basel). 2021 Nov 3;10(11):2369. doi: 10.3390/plants10112369 (PMC8625938; doi:10.3390/plants10112369)
Supplement: Supplementary file 1 [file plants-10-02369-s001.zip › Table S3.pdf]

Table S3 – Category wise list of *cis*-elements extracted from 2 Kbp upstream region of *DcAOX2a* using PlantCare and PLACE.

Categories based on functions	Extracted *Cis*-element (Tools used)		Sequence	Specific functions of *cis*-elements
	NewPlace	PlantCARE		
**Cellular Functions**	AACACOREOSGLUB1	-	AACAAAC	Endosperm-specific gene expression
	ACGTOSGLUB1	-	GTACGTG	Endosperm-specific gene expression
	AGATCONSENSUS	-	TTWCCWWWWNNGGWW	Flower-specific gene expression
	BOXIINTPATPB	-	ATAGAA	Organelle specific (plastid) gene expression
	CAATBOX1	-	CAAT	Tissue specific gene expression
	CACTFTPPCA1	-	YACT	Mesophyll-specific gene expression
	CANBNNAPA	-	CNAACAC	Embryo and endosperm-specific transcription
	CARGATCONSENSUS	-	CCWWWWWWGG	Flowering regulation and vernalisation
	CCAATBOX1	-	CCAAT	Flowering regulation
	-	CCGTCC-box	CCGTCC	Growth and development responsive element
	CEREGLUBOX3PSLEGA	-	TGTAAAAGT	Tissue specific promoter
	CIACADIANLELHC	circadian	CAANNNNATC	Circadian control
	-	dOCT	CaCGGATC	Proliferation-coupled and S phase-specific, which confers tissue-specific expression, such as in newly developing meristems
	DOFCOREZM	-	AAAG	Sperm-specific expression during gametophyte development
	DRE1COREZMRAB17	-	ACCGAGA	Late embryogenesis; ABA and drought responsive
	-	GCN4_motif	TGAGTCA	Endosperm specific gene expression
	-	HD-Zip 2	CAATCATTG	Control of leaf morphology development
	L1BOXATPDF1	-	TAAATGYA	Shoot epidermal cell differentiation, plant trichome development, L1 layer-specific gene expression
	MYBCOREATCYCB1	-	AACGG	Cell cycle phase-independent activation; gene expression induction in actively dividing cells
	NODCON1GM	-	AAAGAT	Nodule specific
	NODCON2GM	-	CTCTT	Nodule specific
	NTBBF1ARROLB	-	ACTTTA	Tissue-specific expression and auxin induction
	POLLEN1LELAT52	-	AGAAA	Anther and pollen development
	PRECONSCRHSP70A	-	SCGAYNRNNNNNNNNNNNNNNNHD	Plastid response element
	RHERPATEXPA7	-	KCACGW	Root hair-specific cis-elements
	ROOTMOTIFTAPOX1	-	ATATT	Organ-specific
	SITEIIATCYTC	-	TGGGCY	Organ/Tissue-specific
	-	Skn-1_motif	GTCAT	Endosperm expression
	S1FBOXSORPS1L21	-	ATGGTA	Plastid-related
	TAAAGSTKST1	-	TAAAG	Guard cell-specific gene expression
	TATCCAOSAMY	-	TATCCA	Sugar and hormone regulation
	TGTCACACMCUCUMISIN	-	TGTCACA	Fruit-specific expression
	WUSATAg	-	TTAATGG	Regulation of stem cell population maintenance in shoots and floral meristems; regulation of genes expressed in the central cells of quiescent centers in the root apical meristem

**Stress Response**	ABRELATERD1	-	ACGTG	Dehydration stress and dark-induced senescence
	ACGTATERD1	-	ACGT	Dehydration stress and dark-induced senescence
	AGCBOXNPGLB	-	AGCCGCC	Ethylene-Induced transcription of defense genes
	ANAERO1CONSENSUS	-	AAACAAA	Anaerobically induced
	ANAERO2CONSENSUS		AGCAGC	Anaerobically induced
	-	ARE	TGGTTT	Anaerobically induced
	ASF1MOTIFCAMV	TGACG-motif	TGACG	Biotic and abiotic (e.g. light) stress stimulated; xenobiotic-stress-activated of genes preferentially expressed in root tip meristems; auxin and MeJA responsive
	-	ATCT-motif	AATCTGATCG	Light-responsive
	BIHD1OS	-	TGTCA	Disease resistance responses
	BOXIIPCCHS		ACGTGGC	Light regulation and ABA responsive
	-	Box 1	TTTCAAA	Light responsive
	-	Box 4	ATTAAT	Light responsive
	CACGTGMOTIF	-	CACGTG	Defence-related, ABA induced and gene regulation during somatic embryogenesis
	-	CATT-motif	GCATTC	Light-responsive
	CGACGOSAMY3	-	CGACG	Sugar responsiveness coupled to environmental stress regulation
	CGCGBOXAT	-	VCGCGB	Involved in ethylene, abscisic acid and light perception, known as Ca2+/calmodulin regulatory element
	-	chs-CMA1a	TTACTTAA	Light responsive
	CURECORECR	-	GTAC	Copper and oxygen-responsive
	EBOXBNNAPA	-	CANNTG	Defense-related gene
	ELRECOREPCRP1	Box-W1	TTGACC	Pathogen- and wound-induced signaling
	-	GA-motif	AARGAWGA	Light responsive
	GATABOX	-	GATA	Light responsive, and tissue specific expression
	-	G-Box	CACGWN<br>TAAACGTG<br>CACGTGG	Light responsive
	GCCCORE	-	GCCGCC	Pathogen responsive, ethylene and jasmonate-responsive
	-	GC-motif	CCCCCG	Anoxic specific inducibility
	GT1CONSENSUS	-	GRWAAW	Light responsive
	GT1CORE	GT1-motif	GGTTAA	Light responsive
	GT1MOTIFPSRBCS	-	KWGTGRWAAWRW	Light responsive
	GT1GMSCAM4	-	GAAAAA	Pathogen- and salt induced
	-	HSE	ARAAAATTYS	Heat stress response
	IBOXCORE	-	GATAA	Light responsive
	INRNTPSADB	-	YTCANTYY	Light responsive
	IRO2OS	-	CACGTGG	Iron regulated
	MYBCORE	-	CNGTTR	Water stress (drought tolerance)
	MYB1AT		WAACCA	Dehydration responsive
	MYB2CONSENSUSAT		YAACKG	Dehydration responsive in abscisic acid signaling
	MYCCONSENSUSAT	-	CANNTG	Cold responsive (freezing tolerance)
	OSE1ROOTNODULE	-	AAAGAT	Mycorrhiza and nodulation-response

	PALBOXAPC	-	CCGTCC	Elicitor or light responsiveness
	PREATPRODH	-	ACTCAT	Proline and hypoosmolarity responsive
	QARBNEXTA	-	AACGTGT	Wounding and tensile stress
	REALPHALGLHCB21	-	AACCAA	Light responsive (required for phytochrome regulation)
	SEBFCONSSTPR10A	-	YTGTCWC	Pathogenesis related
	SORLIP1AT	-	GCCAC	Light responsive on root -specific genes
	SORLIP2AT	-	GGGCC	Light responsive (phytochrome A-regulated)
	SURECOREATSULTR11		GAGAC	Sulfur responsive
	-	Sp1	CC(G/A)CCC	Light responsive
	SREATMSD	-	TTATCC	Sugar repressive element (SRE) identified in genes coupled to abiotic stresses and plant hormone-responsive
	TATABOX5	-	TTATTT	Light responsive
	TBOXATGAPB	-	ACTTTG	Light responsive
	-	TC-rich repeats	ATTTTCTTCA	Defense and stress responsive
	-	TCT-motif	TCTTAC	Light responsive
	WBOXNTERF3	-	TGACY	Wounding induced
	-10PEHVPSBD	-	TATTCT	Light responsive
**Hormonal Regulation**	ABRERATCAL	-	MACGYGB	ABRE-related sequence as Ca2+-responsive
	ACGTABREMOTIFA2OSEM	ABRE	ACGTGKC<br>CACTGT<br>TACGTG	Abscisic Acid (ABA) response and dehidratation responsive (DRE)
	ARFAT	-	TGTCTC	Auxin-responsive
	ARR1AT	-	NGATT	Cytokinin-regulated
	CPBCSPOR		TATTAG	Cytokinin-regulated
	DPBFCOREDCDC3	-	ACACNNG	ABA-responsive and embryo-specification elements
	-	ERE	ATTTCAAA	Ethylene-responsive
	ERELEE4	-	AWTTCAAA	Ethylene responsive
	GAREAT		TAACAAR	GA-responsive
	-	GARE-motif	AAACAGA	GA-responsive
	GARE1OSREP1	-	TAACAGA	GA-responsive
	MYBGAHV		TAACAAA	GA-responsive
	PROXBBNNAPA		CAAACACC	ABA-responsive
	PYRIMIDINEBOXHVEPB1		TTTTTTCC	GA and abscisic acid responsive
	PYRIMIDINEBOXOSRAMY1A		CCTTTT	Induced in the aleurone layers in response to GA
	RYREPEATBNNAPA		CATGCA	ABA-responsive
	T/GBOXATPIN2		AACGTG	Jasmonate-signaling
	WBOXATNPR1		TTGAC	SA-induced pathogenesis-defence
	WRKY71OS		TGAC	GA signaling pathway; ABA signalling; plant defence signalling
