# Supplementary material for: Carrot AOX2a Transcript Profile Responds to Growth and Chilling Exposure
Source: Plants (Basel). 2021 Nov 3;10(11):2369. doi: 10.3390/plants10112369 (PMC8625938; doi:10.3390/plants10112369)
Supplement: Supplementary file 1 [file plants-10-02369-s001.zip › Table S4.pdf]

Table S4. Primers used in RT-qPCR. AS corresponds to amplicon size and E corresponds to primer efficiency.

Gene	Primer sequence (5'→3')	AS (bp)	E (%)
EF-1A	Fw: TGGTGATGCTGGTTTCGTTAAG<br>Rv: AGTGGAGGGTAGGACATGAAGGT	75	99
GAPDH	Fw: GGGAGGTGCAAAGAAAGTTATCA<br>Rv: TTCCTTTTCATTGACACCAACAA	79	96
5.8S	Fw: AATGACTCTCGGCAACGGATAT<br>Rv: TCACACCAAGTATCGCATTTCG	73	102
DcAOX1	Fw: CTTCAACGCCTACTTCCTTG<br>Rv: ATCTCGCAATGTAGAGTCAGC	196	97
DcAOX2a	Fw: TCTTCAATGCTTTCTTTGTTCTT<br>Rv: GACATCTTTTAGTTTGGCATCTTT	200	93

AS: amplicon size; E: primer efficiency.
