# Supplementary figures and images for: Carrot AOX2a Transcript Profile Responds to Growth and Chilling Exposure
Source: Plants (Basel). 2021 Nov 3;10(11):2369. doi: 10.3390/plants10112369 (PMC8625938; doi:10.3390/plants10112369)

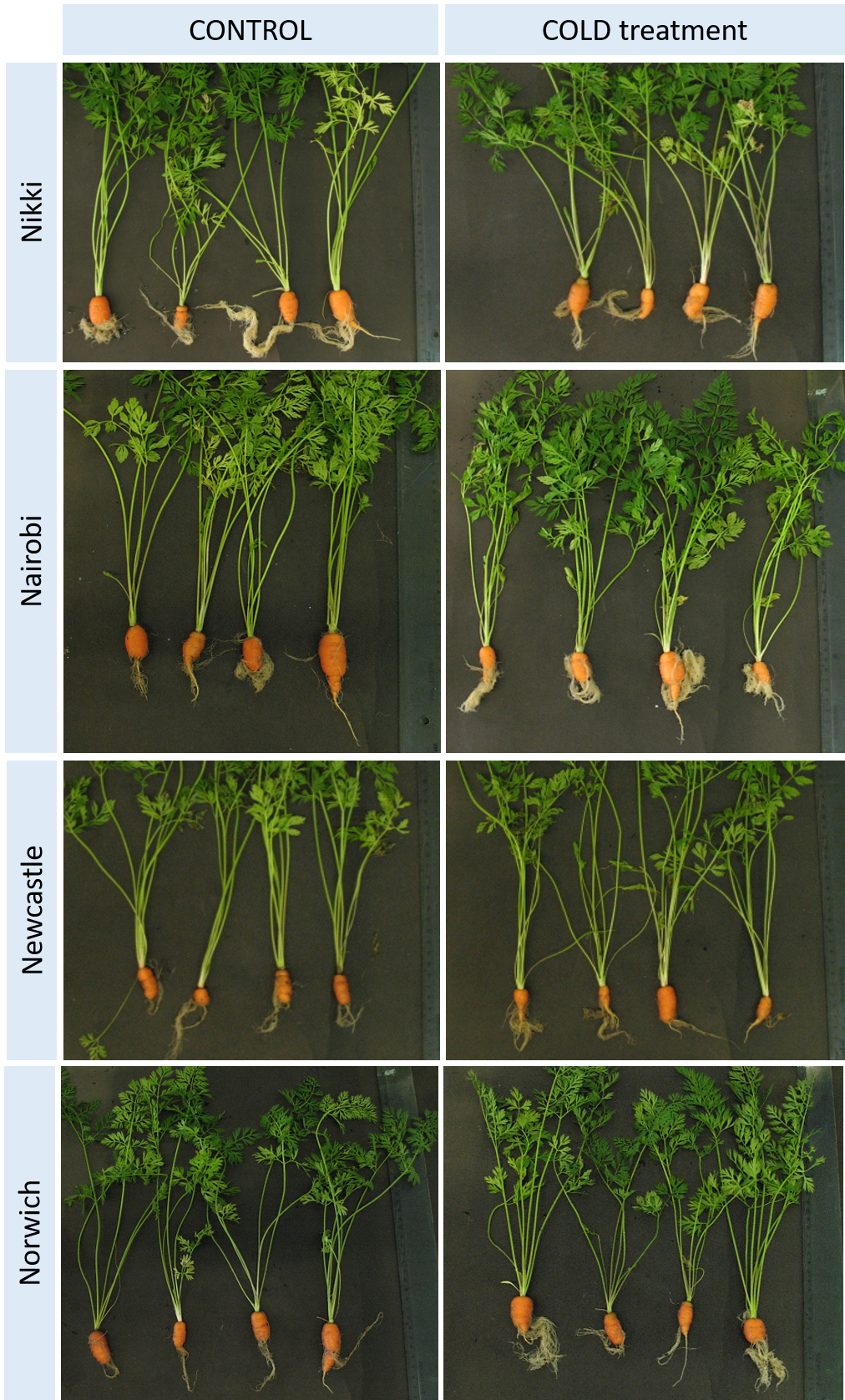

Supplement: Supplementary file 1 [file plants-10-02369-s001.zip › Figure S1.tif]
